# Application of extracellular vesicles in diabetic osteoporosis

**DOI:** 10.3389/fendo.2024.1466775

**Published:** 2024-12-10

**Authors:** Xiaopeng Jia, Gongzi Zhang, Deshui Yu

**Affiliations:** ^1^ Trauma Orthopedics, The First Affiliated Hospital of Jinzhou Medical University, Jinzhou, China; ^2^ Department of Rehabilitation Medicine, Chinese People’s Liberation Army General Hospital, Beijing, China

**Keywords:** diabetic osteoporosis, extracellular vesicles, osteoblasts, osteoclasts, oxidative stress

## Abstract

As the population ages, the occurrence of osteoporosis is becoming more common. Diabetes mellitus is one of the factors in the development of osteoporosis. Compared with the general population, the incidence of osteoporosis is significantly higher in diabetic patients. Diabetic osteoporosis (DOP) is a metabolic bone disease characterized by abnormal bone tissue structure due to hyperglycemia and insulin resistance, reduced bone strength and increased risk of fractures. This is a complex mechanism that occurs at the cellular level due to factors such as blood vessels, inflammation, and hyperglycemia and insulin resistance. Although the application of some drugs in clinical practice can reduce the occurrence of DOP, the incidence of fractures caused by DOP is still very high. Extracellular vesicles (EVs) are a new communication mode between cells, which can transfer miRNAs and proteins from mother cells to target cells through membrane fusion, thereby regulating the function of target cells. In recent years, the role of EVs in the pathogenesis of DOP has been widely demonstrated. In this article, we first describe the changes in the bone microenvironment of osteoporosis. Second, we describe the pathogenesis of DOP. Finally, we summarize the research progress and challenges of EVs in DOP.

## 1 Introduction

Osteoporosis is a systemic bone disorder characterized by low bone mass and deterioration of the microstructure of bone tissue, often resulting in bone fragility and an increased risk of fractures (1). Osteoporosis leads to fragility fractures, with a significantly increased risk of fractures of the proximal femur and distal radius, with vertebral compression being the most common (2). Globally, osteoporosis causes more than 8.9 million fractures each year (3). Osteoporosis occurs primarily in older patients, affecting more than 14 million people in the United States and more than 200 million people worldwide (4, 5). According to the International Osteoporosis Foundation, osteoporosis is the leading cause of fracture in older adults in patients over the age of 50 (6). Osteoporotic fractures are more common in women than in men, with 1 in 3 women and 1 in 5 men at the risk of osteoporotic fractures. This number continues to rise as the population ages (7). It is estimated that the annual economic consumption of osteoporosis in the United States alone will climb to $25.3 billion by 2025 (8). Osteoporosis leads to a reduced quality of life for patients, and the financial burden of medical expenses for its treatment is significant. Clearly, osteoporosis has become a global public problem.

Factors such as genetics, hormone deficiencies, and aging can all contribute to disruption of bone homeostasis maintained by osteoblasts and osteoclasts, leading to osteopenia and deterioration of the microstructure of bone tissue, ultimately leading to osteoporosis (9). Diabetes is one of the main causes of osteoporosis. Studies have shown that the incidence of osteoporosis in diabetic patients is 60%, which is significantly higher than that of non-diabetic patients (10). Diabetes mellitus leads to abnormal blood glucose metabolism, abnormally elevated levels of extracellular metabolites, and disruption of bone remodeling (11). Diabetic osteoporosis (DOP) is a systemic chronic metabolic bone disease characterized by increased cortical porosity, imbalance of bone metabolism, and distortion of bone microstructure (12). DOP is often associated with osteoporotic fractures due to abnormal glucose metabolism, accumulation of advanced glycation end products (AGEs), and damage to the osteovascular system (13). Although the specific mechanism of DOP has not yet been clarified, many reports have confirmed the relationship between the two (14). At present, the clinical treatment of osteoporosis is mainly based on bisphosphonate, alendronate sodium inhibition of bone resorption capacity of osteoclasts, osteotriol and calcium and other bone drugs, and drugs that promote osteoblast function (15–17). Despite the success of these treatments, the incidence of fractures due to DOP remains high.

**Figure 1:**
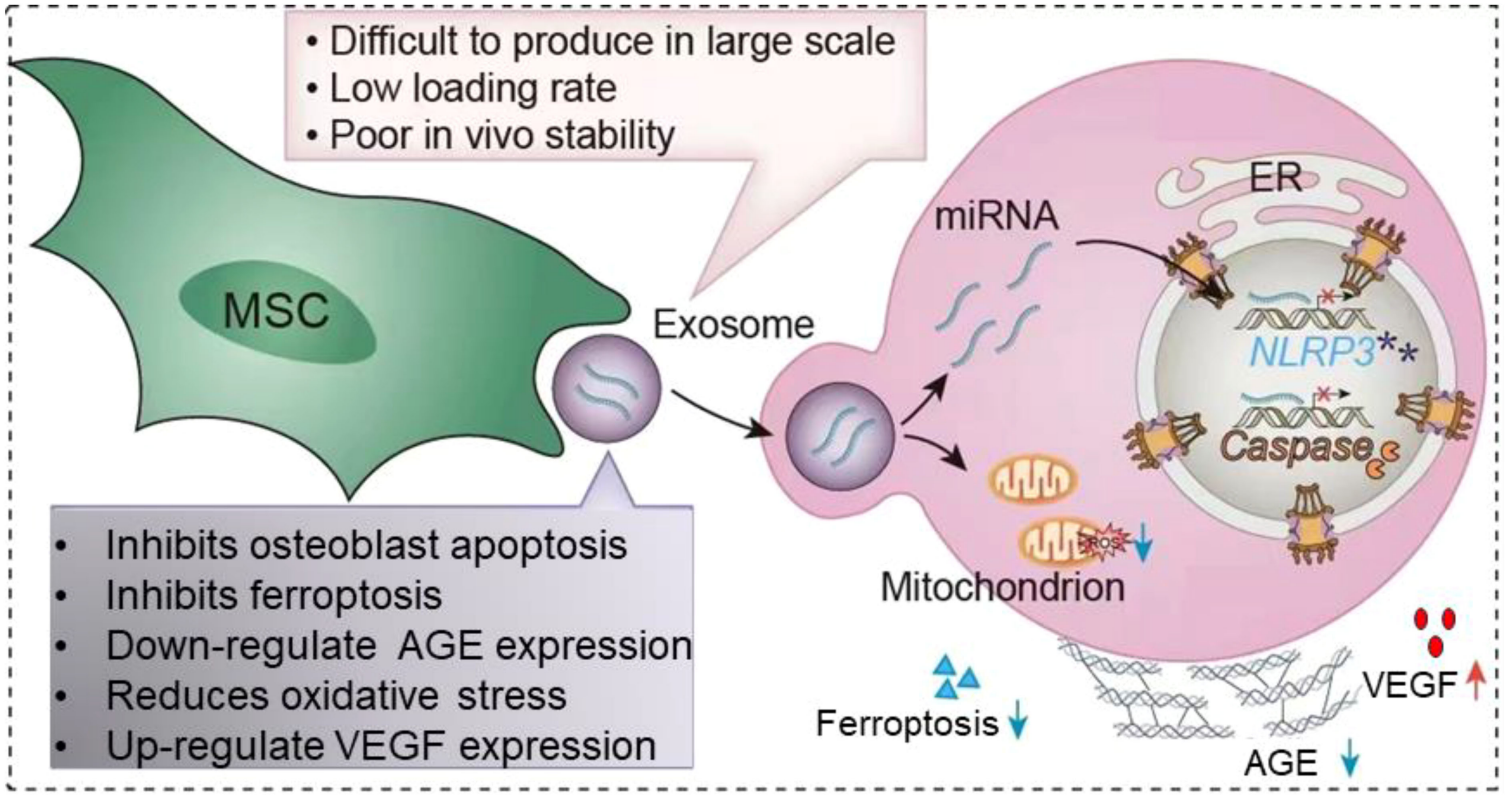
Application of extracellular vesicles in diabetic osteoporosis. MSC-EVs is able to be captured by target cells via endocytosis. EVs are able to carry the miRNAs and proteins they carry into target cells. It leads to decreased apoptosis of osteoblasts, inhibits ferroptosis, downregulates AGE, reduces oxidative stress, promotes VEGF secretion and thus promotes angiogenesis. However, the clinical application of EVs is faced with factors such as how to mass produce in large quantities, low loading rates and poor stability *in vitro*. The red cross means represents inhibition, and purple asterisk means NLRP3 post-catabolic products.

At present, extracellular vesicles (EVs) have attracted attention as a special way of signaling between cells (18). EVs have a double-layer lipid membrane structure, which has the advantages of low immunogenicity and good cell compatibility *in vivo* (19). EVs are vesicles produced by paracrine in living cells, and EVs can deliver miRNAs and proteins in cells to target cells through membrane fusion, and regulate the biological activity of target cells (20). The role of EVs in DOP has been well established (**Figure 1**). In this article, we first describe the changes in the osteoporotic bone microenvironment. Second, we describe the pathogenesis of DOP. Finally, we summarize the research progress and challenges of EVs in DOP.

## 2 Osteoporotic microenvironment

Bone provides a physical scaffold for the daily activities of vertebrates. The cells in the bone mainly include osteoblasts, osteoclasts, and bone marrow mesenchymal stem cells (21). Osteoblasts are the main cells responsible for bone formation, and these cells secrete cell matrices such as type I collagen, osteopontin, osteocalcin, and alkaline phosphatase (22). Osteoclasts play a major role in the body for bone resorption (23). Bone has a unique ability to regenerate and repair, and the regulation of the balance of osteoblasts and osteoclasts is the basis for this unique repair ability (24). The stem cells in bone are mainly bone marrow mesenchymal stem cells and bone stem cells (25). Bone marrow mesenchymal stem cells (BMSCs) form other cells in the bone microenvironment (ECM), including osteoblasts, chondroblasts, adipocytes, and other stromal cells (26). The ECM regulates the biological behavior of cells, providing biochemical signals to cells and physical and mechanical signals to cells (27). The ECM is mainly composed of type I collagen fibers (85-90%), and hydroxyapatite, as a component that enhances the mechanical properties of collagen fibers, can increase the tensile modulus of collagen matrix by 4 times (28). In addition to collagen fibers, ECM also contains a large amount of non-collagen components such as osteocalcin, osteopondin, osteopontin, adhesion proteins, and proteoglycans (29). Non-collagen components are not only involved in the composition of the ECM, but also play an integral role in the bone remodeling process (30). Bone remodeling is the dynamic equilibrium process of bone ECM, which occurs during a person’s lifetime activities. Bone remodeling can be separated into five phases: activation; resorption; reversal; formation; and termination (31). Abnormal bone remodeling, which leads to a decrease in bone mass, density, mass, and strength, is the most important factor in osteoporosis (32).

**Figure 2:**
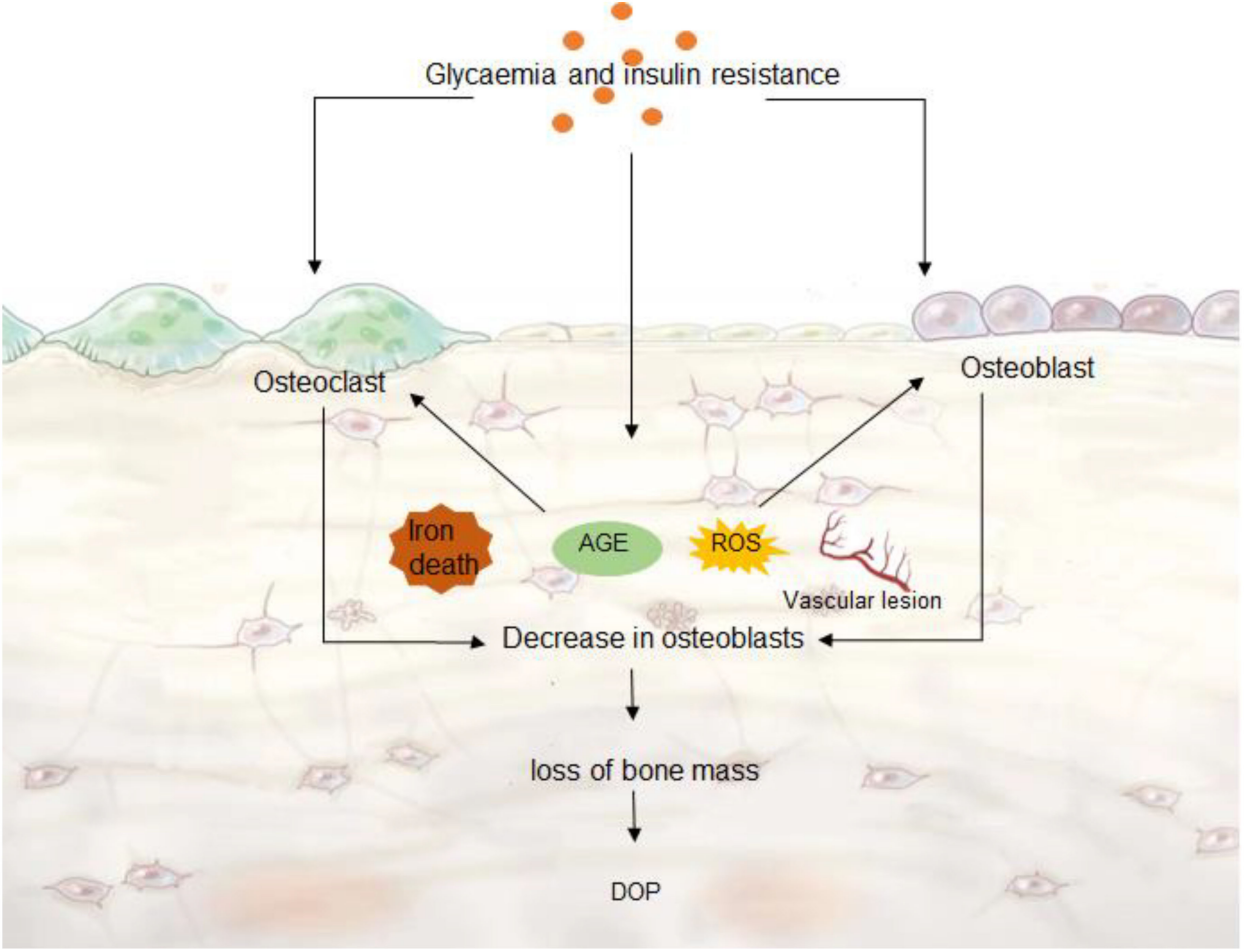
DOP microenvironment. Hyperglycemia and insulin resistance can cause ferroptosis, ROS and AGE production, vascular lesions, which further accelerate the decrease of osteoblast activity, and the enhancement of osteoclast activity leads to abnormal bone remodeling. Decreased bone cell content leads to DOP.

Osteoblasts are derived from BMSCs *in vivo* and are thought to be the only cells in vertebrates that can promote bone regeneration (33). BMSCs are pluripotent cells in the bone marrow that can differentiate into osteoblasts, adipocytes, and chondrocytes (34). Differentiation of BMSCs into osteoblasts is first required to induce Runx family transcription factor 2 (RUNX2) (35). 60% to 80% of osteoblasts differentiated by BMSCs undergo apoptosis, and the remaining osteoblasts become mature osteoblasts (36). Osteoblasts secrete extracellular matrix proteins, type I collagen, and other non-collagen proteins (37).ECM is first secreted in an unmineralized osteoid form and subsequently mineralized into bone by the accumulation of calcium phosphate in the form of hydroxyapatite (38). In addition, osteoblasts promote osteoclast maturation and proliferation. Osteoblasts secrete macrophage colony-stimulating factor (M-CSF) that binds to the colony-stimulating factor-1 receptor (c-FMS) in osteoclasts, thereby activating phosphoinositide 3-kinase (PI3K) and growth factor receptor-binding protein 2 (Grb2) and further promoting osteoclast differentiation and maturation (39). Osteoclasts arise from the bloodline monocyte-macrophage system and are the primary functional cells for bone resorption (40). Factors such as age, gender, trauma, and medications can all lead to disruption of bone remodeling balance, dysfunction of bone cell activity and abnormal cytokine secretion, which ultimately leads to osteopenia and bone imbalance (41). The decrease in collagen fibers and bone mineral salt content, the reduction, fracture or disappearance of trabeculae leads to the relaxation of bone tissue structure, which in turn affects the mechanical nature of the skeletal system and leads to fractures (42). The changes in the osteoporosis microenvironment are mainly caused by the destruction of bone remodeling balance caused by the decrease of osteoblast activity and the enhancement of osteoclast activity, which in turn leads to the decrease in the number of osteocytes and the occurrence of osteoporosis (**Figure 2**).

## 3 Pathogenesis of DOP

### 3.1 Hyperglycemia and insulin resistance

The main factors of diabetes mellitus are insulin deficiency due to decreased function of β cells in the pancreas to secrete insulin, or insulin resistance due to decreased physiological effects of insulin in cells in the body due to insulin insensitivity leading to insulin-related physiological effects (43). Insulin resistance and chronic inflammatory processes go hand in hand (44). Adipose tissue releases IL-1β, IL-6, and TNFα by activating M1 macrophages, creating a pro-inflammatory environment that blocks the action of insulin (45). The release of inflammatory factors exacerbates aberrant activation of nucleotide-binding oligomeric domain-like receptor protein 3 (NLRP3) inflammasomes, and autoinflammatory mechanisms may exacerbate pancreatic injury (46). Insulin resistance may affect bone health by impairing the insulin-like growth factor-1 (IGF-1) signaling pathway (47). Insulin inhibits the expression of IGF-binding protein-1 (IGFBP-1) in osteoblasts, and insulin deficiency increases IGFBP-1 levels, thereby reducing the anabolic effects of unbound IGF-1 in bone (48). In addition, studies have shown that reduced nitric oxide (NO) bioavailability is one of the mechanisms of diabetes-induced osteoporosis (49). Endoxela Nocintas (Enos) de Rivedno can reduce osteoclast activity, thereby reducing bone destruction and reducing the incidence of osteoporosis (50). Insulin resistance can also eventually lead to an increase in glucose in the body.

Glucose is the main source of bone development and growth and plays a vital role in the skeletal system (51). Glycolysis plays an important role in osteoclast differentiation. Blocking the glycolytic pathway has been shown to inhibit osteoclast production (52). Mature osteoclasts have been shown to have higher rates of glycolysis (40). Diabetes disrupts the balance of bone metabolism and can lead to osteoporosis (53). Glucose stimulates the non-canonical Wnt/protein kinase C pathway and upregulates the peroxisome proliferator-activated receptor γ (PPARγ), resulting in increased BMSC differentiation to adipose and bone loss (54). While some adipogenic factors such as adiponectin, endolipids, and omentin-1 have negative effects on bones (55). Not only that, higher blood glucose levels in type 2 diabetes are inversely correlated with serum osteocalcin levels (56). Osteocalcin is considered a biochemical marker of bone remodeling, and a hallmark of type 2 diabetic bone disease is decreased bone formation and decreased serum osteocalcin (57). In addition, hyperglycemia can lead to osmotic diuresis, impaired reabsorption of calcium ions in the renal tubules and collecting ducts, which in turn leads to a decrease in blood calcium levels (58). Decreased serum calcium stimulates parathyroid hormone secretion, leading to increased osteoclast activity, which in turn exacerbates bone destruction (59).

Insulin resistance and hyperglycemia have been widely demonstrated in the pathogenesis of TOP. Control of blood glucose levels reduces the incidence of DOP. Therefore, insulin resistance and hyperglycemia have a non-negligible role in the occurrence of DOP.

### 3.2 Oxidative stress

Reactive oxygen species (ROS) are normal metabolites of oxygen, and when they are produced in excess, they can lead to a disruption of the oxidant-antioxidant balance (60). Diabetic patients have an increased release of oxidative factors in the body due to disorders in blood glucose metabolism (61). Studies have shown that biomarkers of oxidative stress are positively correlated with glucose concentration, and antioxidant enzymes are negatively correlated with glucose concentration (62). Hyperglycemia can lead to excessive production of ROS, which is not only a major factor in aging, but also one of the leading causes of diabetes complications, insulin resistance, and mitochondrial dysfunction (63). The massive accumulation of ROS can lead to protein inactivation and lipid membrane peroxidation, which can lead to DNA damage (64). Not only that, the accumulation of a large amount of ROS can also lead to the inactivation of various enzyme systems in the body, which in turn will disrupt the normal level of substance metabolism in the body (65). In addition, excess ROS can alter the microenvironment of osteoblasts, destroy bone microstructure, reduce bone formation, and prolong fracture healing time (66). Wang et al. showed that hyperglycemia and oxidative stress can exacerbate the accumulation of advanced glycation and glycooxidation end products (AGEs/AGOEs) in bones (67). The increase in AGEs/AGOEs in the bone microenvironment leads to increased bone fragility and thus osteoporosis (68). Zhou et al. showed that the expression level of oxidative stress-related genes in osteoporosis patients was significantly higher than that of normal people, such as Supero Hidededis, Mutassé (Saud), Ineris Rosites, Catalas (Kate), Totar, Antio, Hitant, Status (Tass), Andesidro Prosides (Sea) (69). Therefore, oxidative stress plays an important role in the development of osteoporosis.

In bone metabolism, ROS mainly relies on mitochondria, xanthine oxidase and NADPH oxidase to promote osteoblast apoptosis and inhibit osteoblast differentiation (70). In addition, ROS promotes osteoclast differentiation mainly by inducing multinuclear fusion of BMSCs and increasing the expression of NFATc1, CTSK and MMP9 through MAPK and NF-κB signaling pathways (71). Therefore, the accumulation of ROS can cause bone healing disorders and promote bone aging (72). Jiang et al. confirmed that inhibition of autophagy and oxidative stress *in vivo* can reduce the incidence of DOP (73). ROS not only affects osteogenic differentiation but also stimulates osteoblast apoptosis (74). Reducing inflammatory oxidative stress can reduce the occurrence of DOP.

Hypoxia-inducible factor (HIF-1) is the main regulator of hypoxia adaptation response, and as a transcription factor, HIFs are involved in maintaining cellular oxygen homeostasis (75). Hyperglycemia promotes ubiquitination of the carboxy-terminus of HIF-1α ligase and subsequent proteasomal degradation, thereby destabilizing HIF-1α (76). Wang et al. showed that SENP3 overexpression inhibited osteoclast differentiation in type II DOP by increasing the stability and expression of HIF-1α (77). Metformin (Met) is a potent anti-diabetic and redox modulator (78). Met has been shown to improve insulin resistance and oxidative stress levels in type 2 diabetes through the Keap1/Nrf2/Ho-1 pathway (79). Chen et al. confirmed that Met can inhibit hyperglyce-induced oxidative stress by activating the Nrf2/HO-1 signaling pathway in type 2 DOP, reducing bone loss and bone microstructure destruction (72). In addition, Jiang et al. also confirmed that inhibition of oxidative stress can enhance collagen fibers, changes in trabecular bone density, and improve changes in bone microenvironment (73).

Oxidative stress has been shown to accelerate the progression of degenerative diseases. Preclinical studies have reported a large number of studies in which reducing oxidative stress and delaying the progression of DOP have been reported. Moreover, oxidative stress is one of the initiating factors affecting the DOP microenvironment. Reducing oxidative stress can not only maintain the cellular activity of osteoblasts, osteoclasts and BNSCs, but also reduce bone loss and damage to bone microstructure, and delay the progression of DOP. Therefore, reducing oxidative stress plays an important role in the occurrence of DOP.

### 3.3 Ferroptosis

Iron participates in various biochemical processes such as enzymatic reactions and oxygen transport in the body, and is one of the indispensable trace elements in human life (80). Iron enters cells by binding to transferrin receptor 1 (TFR1) on the cell membrane to reduce ferric iron to iron (81). Subsequently, divalent metal transporter 1 (DMT1) releases ferric iron into the labile iron pool (LIP) in the cytoplasm. Lysosomes recycle ferritin and iron from mitochondria (81, 82). Ferroptosis is a form of apoptosis that relies on intracellular iron, and iron overload caused by abnormal iron metabolism is one of the main features of ferroptosis (83). Iron overload decreases cell viability, glutathione, and superoxide dismutase levels, increasing ROS production and lipid peroxidation (84). Iron can reach the mitochondrial matrix through the outer mitochondrial membrane and inner mitochondrial membrane, where it subsequently regulates the physiological functions of important organelles in the mitochondria (85). Diane et al. confirmed that mitochondrial transferrin 1/2 (Mfrn1/2) on the inner mitochondrial membrane is destroyed, resulting in abnormal mitochondrial iron metabolism (86). Although the specific mechanism of ferroptosis-induced osteoporosis is elusive, the occurrence of ferroptosis and osteoporosis has been widely confirmed (87).

Ferroptosis relies primarily on iron overload and lipid peroxidation (88). Biological features of ferroptosis include decreased cystine uptake, reduced glutacystine depletion, inhibition of cystine/glutamate reverse transport system (Xc system) activity, and aberrant accumulation of iron ions and ROS (12). Ferroptosis can also lead to the breakdown of the barrier between bone resorption and bone formation, which can lead to osteoporosis (89). Iron overload-mediated oxidative stress increases ROS levels, impairs mitochondrial dysfunction, and decreases the mineralization and osteogenic differentiation capacity of BMSCs (90). Hyperglycemia can cause overexpression of divalent metal transporter 1 (DMT1) in osteoblasts, which can induce ferroptosis (91). Ma et al. demonstrated that melatonin inhibits hyperglyce-induced ferroptosis by activating the Nrf2/HO-1 signaling pathway in type 2 DOP, thereby delaying the onset of osteoporosis (89). Nuclear factor erythroid-2 related factor 2 (NRF2) is an antioxidant stress gene *in vivo* that regulates oxidative stress by controlling the expression of heme oxygenase-1 (HO-1) and a variety of other proteins with antioxidant and anti-inflammatory effects (92). Zhang et al. confirmed that activation of NRF2 can inhibit hyperglucose-induced ferroptosis in osteoblasts, thereby reducing the occurrence of osteoporosis (93). Lin et al. demonstrated that hyperglycemia induces ferroptosis (upregulation of ferroptosis-related genes SLC7A11 and GPX4 expression in osteoporoblasts by activating the METTL3/ASK1-p38 signaling pathway, resulting in osteoporosis (94). Ferroptosis has been widely demonstrated in the occurrence of DP (95–97).

### 3.4 Build-up of advanced glycation products

In the hyperglycemic state, non-enzymatic glycosylation of proteins, phospholipids, and nucleic acids (whose classical pathway is also known as the Maillard reaction) leads to the formation of advanced glycation end products (AGEs) (98). Because of its higher surface-to-volume ratio, AGE accumulates significantly higher in trabecular bone than in cortical bone (67). AGE leads to decreased bone formation by decreasing osteoblast differentiation and proliferation, downregulating matrix production, and increasing osteoblast apoptosis (99). Studies have shown that AGEs not only induce osteoclast production by upregulating RANKL mRNA, but also affect osteoblasts by inhibiting cell growth, promoting apoptosis, and downregulating differentiation (100). Elevated blood sugar in diabetic patients can induce excessive accumulation of AGEs in bones, which in turn leads to osteoporosis (101). Moreover, studies have found that pathological activation of AGE and AGE receptor (RAGE) signaling inhibits the osteogenic differentiation of BMSCs by modulating DNA methylation and Wnt signaling (102). Inhibition of AGE-RAGE activation can reduce the occurrence of DP (103). In addition, activation of RAGE enhances apoptosis, inflammation, and oxidative stress that directly damages cells and tissues (104). Notsu et al. showed that AGE-RAGE increased TGF-β expression and secretion and inhibited stromal cell mineralization (105). Ding et al. demonstrated through *in vivo* experiments in mice that the number of osteoclasts decreased, bone resorption decreased, bone mass increased, biomechanical strength was improved, and the occurrence of cancellous femoral bone was reduced in mice that were knocked out or inhibited (106). Inhibition of RAGE signaling is to improve the ability of BMSCs to differentiate into osteoblasts and osteocytes under diabetic conditions (100).

The study of AGE in DOP has gradually been recognized. AGE affects trabecular bone distribution in the bone microenvironment, alters bone mineral density, and leads to DOP. Reducing the expression of AGE can inhibit osteoclast acquisition and delay the progression of AD. Reducing the expression of AGE plays an important role in the occurrence of DOP.

### 3.5 Osteovascular

Hyperglycemia can lead to microvascular and macrovascular lesions. Angiogenesis in bone and its role in bone metabolism fundamentally affect the pathogenesis of osteoporosis (107). Neovascularization is closely related to osteogenic activity, which not only directly provides oxygen, nutrients and growth factors to the osteogenic active area, but also promotes osteogenic activity by secreting vascular secretion signals to create a microenvironment suitable for the pre-survival of mesenchymal stem cells and osteoblasts (108). There is a capillary subtype in the bone that is involved in bone formation and stimulates the proliferation of bone progenitor cells and differentiation factors within the bone marrow to actively guide bone formation, and this blood vessel is known as H-shaped capillaries (109). Reduction of H-type capillaries is associated with bone loss in a mouse model of aging and postmenopausal osteoporosis (110). Hu et al. found that damage to H-type blood vessels by NADPH oxidase 2 (NOX2)-mediated endothelial oxidative stress may be a target for type 1 diabetes-induced bone disease (111). Activation of Notch and Hif-1α signaling pathways in bone tissue and exogenous PDGF-BB treatment increased the number of H-type blood vessels while restoring bone mass (112). Type H capillary injury is associated with a variety of factors. Lectin-like oxidized low-density lipoprotein receptor 1 (LOX-1) is a major receptor for the recognition of oxidized LDL and is involved in lipid metabolism and the development of vascular inflammation (113). LOX-1 can promote osteoclast activity and enhance bone destruction (114). Qiu et al. confirmed that knockdown of LOX-1 gene can enhance bone mineral structure and improve the expression of trabecular microstructure and osteogenesis-related genes such as bone morphin 2 (BMP2) and osteopontin (OPN) (115). βII-Spectrin (SPTBN1) is an actin cross-linked molecular scaffolding protein involved in the arrangement of transmembrane proteins and organelle organization *in vivo* (116). Xu et al. found that SPTBN1 could not regulate osteoblast differentiation through TGF-β/Smad3 and STAT1/Cxcl9 signaling pathways, and could also promote the expression of vascular endothelial growth factor (VEGF) to promote the formation of skeletal blood vessels (117).

Blood vessels maintain the transition between nutrients and metabolic wastes in bone tissue, providing growth essentials for the bone microenvironment. Bone tissue is inseparable from the support of blood vessels. In the DOP setting, the generation of new blood vessels is a key factor in reversing disease progression.

## 4 Application of extracellular vesicles in DOP

### 4.1 EVs

EVs are a heterogeneous population of membrane vesicles secreted by living cells and play an important role in maintaining cellular function and tissue homeostasis in multicellular organisms (118). According to their subcellular origin and size, they are divided into three main groups: apoptotic bodies, membrane vesicles, and EVs. EVs are membrane-vesicle-like structures with a diameter of about 30-150 nm, which contain proteins, lipids, and RNA in cells (19, 119). EVs originate from nanoscale vesicles formed by inward budding from endosomal membranes and are secreted into the extracellular environment when membrane vesicles fuse with the plasma membrane (120). As a special way of information transmission between cells, Evs can deliver the goods carried by itself to target cells through endocytosis and cytocytosis, thereby regulating the life process of target cells (119). Because EVs are produced by living cells and have a double-layer lipid membrane structure, EVs have the advantages of low immunogenicity and good cell compatibility *in vivo* (19). In addition, EVs can also transfer the carnage into the EVs through exogenous loading pathways, such as co-incubation, thermal shock, sonication, and electroporation (121). The advantage of EVs is that they can transport the contents to target cells without being degraded (122). In addition, chemical modifications give EVs unique targeting capabilities (123). EVs from different cell sources have different targeting abilities *in vivo* (124). This type of cell homing is species-independent. Chemical modification and genetic engineering are the main improvement methods for EV membrane modification (125). For example, Alvarez-Erviti et al. targeted the central nervous system by genetically engineering dendritic cells to express Lamp2b and RVG peptides (126).

The ability of stem cells to self-proliferate and differentiate in a directed manner plays an important role in tissue engineering and regenerative medicine (127). However, dead cells produced by stem cells after local metabolism can trigger local inflammation and tumorigenesis (128). Exogenous stem cells are not conducive to survival and proliferation in the ischemic and inflammatory microenvironment, and may even lead to the death of many cells in the body (118). Not only that, but the retention and undersurvival of mesenchymal stem cells at the site of administration can hinder their therapeutic efficacy (129). EVs can avoid the toxic side effects of cell therapy. EVs are paracrine production that not only maintain part of the function of the cell from which they originate, but can fuse with the cell membrane of the target cell after it has functioned (130). The role of EVs has been proven in many areas, such as tumors, obligatory spondylitis, diseases of the cardiovascular system, osteoarthritis, etc (131–134).

### 4.2 Application of EVs in DOP

The use of EVS in DOP has been demonstrated in preclinical studies and is considered a meaningful therapeutic trend for DOP. Adipose tissue-derived MSCs have shown strong potential in reducing inflammation in the body and improving the paracrine effects of chondrocytes (10). Tofino et al. demonstrated that adipose tissue-derived MSCs (AD-MSCs)-derived EVs significantly reduce inflammatory mediators and inhibit nitric oxide synthase activity in osteoarthritis chondrocytes (135). Decreased NO bioavailability is one of the mechanisms of diabetes-induced osteoporosis (49). Therefore, can EVs derived from AD-MSCs also play a role in DOP? To verify this effect, Zhang et al. isolated AD-MSCs-derived EVs and confirmed that they can alleviate diabetic osteoporosis in mice by inhibiting NLRP3 inflammasome activation in osteoclasts in diabetic mice (136). AD-MSCs-derived EVs inhibit the production of pro-inflammatory cytokines IL-1β and IL-18 and inhibit the production of apoptotic factor caspase-1 for 72 hours *in vivo*. Moreover, AD-MSCs-derived EVs reduced the loss of bone mineral content and the loss of bone mineral density at day 28 in mice. Zhang et al. transduced miR-146a into AD-MSCs-derived EVs *in vitro* to enhance its anti-inflammatory effect and delay the progression of DOP (137). miR-146a can modulate inflammation and osteogenesis (138). AD-MSCs-derived EVs overexpressing miR-146a could significantly inhibit NLRP3 inflammasome activation and alleviate the pathogenesis of DOP. Therefore, EVs can reduce inflammation in DOP and promote osteoblast activity, delaying the progression of LOP.

Abnormal macrophage polarization and pro-inflammatory M1 macrophage hypersensitivity can be detected in DOP. Appropriate interventions to promote macrophage polarization to the M2 subtype will help promote diabetic fracture healing (139). Wang et al. cultured M2 macrophages to produce EVs that induce the transformation of M1 macrophages into M2 macrophages by stimulating the PI3K/AKT pathway (140). M2 macrophages increase bone morphogenetic protein 2 (BMP-2) expression, leading to cartilage and bone formation. Bone marrow mesenchymal stem cells (BMSCs) have the potential for multidirectional differentiation and self-renewal, playing a central role in bone homeostasis and regeneration (141). Studies have shown that during the process of cellular senescence, BMSCs tend to differentiate into adipocytes, resulting in low bone mass and bone marrow fat accumulation (142). MSC-derived exosome metastases miR-146a, miR-126, miR-128-3p, and miR-196a, among others, to alleviate DOP and reduce the incidence of fractures (143). It was found that the elevated expression of miR-382-3p and miR-550a-5p in the serum of patients with type 2 diabetes affected the osteogenic differentiation of BMSCs (144). miR-221, as one of the highly expressed miRNAs in diabetic BMSC exosomes, has the ability to inhibit osteogenesis and promote adipogenesis (145). Han et al. showed that inhibition of miR-221 expression in BMSC-EVs increased bone mass, reduced bone marrow fat accumulation, and promoted bone regeneration in diabetic mice (143).

EVS acts as a carrier to transfer the cargo to the osteoporosis microenvironment, which delays the progression of osteoporosis under the premise of protecting the carrier from destruction. Although studies of Evs in osteoporosis have been widely reported, there are few preclinical studies of EVs in DOP. The therapeutic role of EVs in DOP has been confirmed in all published preclinical studies.

### 4.3 Challenges in the application of EVs in DOP

The most important factors for EVS are how to stabilize production on a large scale and have low load efficiency (118). Despite the multiple advantages of EVs, there are still no standardized recommendations on how to produce, purify, and store them on a large scale (146). In a laboratory setting, less than 1 ug of exosomal protein should only be harvested from 1 ml of medium (147). Although there are many biochemical strategies, physical strategies (hypoxia, heat stress, and starvation), mechanical strategies (shear stress and 3D culture), and instrumental strategies, among others (148). However, due to the differences in size, surface marking, content, and origin of EVs, separating them is also a major challenge (149). Grangier et al. reported a 3D incubation platform that can minimize operation time, incubation cycle, and cost to enable large-scale production of EVs (150). However, mass-produced Evs also need to undergo ultracentrifugation for purification. Although ultracentrifugation is the gold standard for the separation and purification of EVs (151). However, the purification of EVs is limited by the time-consuming, costly, inefficient, and lipoprotein co-separation of large-scale EVS (149). In recent years, immunoaffinity chromatography, antigen/protein conjugation, ultrafiltration, and size exclusion chromatography have all been used for mass production of EVs (146). While no single technique is perfect, combining the above techniques with others may be the solution to meet multiple requirements for exosome batch production and purification simultaneously (151).

In addition, the low loading efficiency of EVs and how they are chemically modified to be targeted are another factor limiting their application (152). The modification of exosomes is divided into internal strategies (drug loading) and external strategies (surface modifications). The primary purpose of surface modification is to selectively deliver exosomes to target cells for precise processing (153). Although co-incubation, sonication, thermal shock, and electroporation can improve the loading rate, their effect is limited (154). For example, the loading efficiency of co-incubation is low, and ultrasonic electroporation disrupts the membrane structure of EVs (155). In addition, the long-term storage of EVs is also a limiting factor for their application. At present, the methods used for long-term storage of EVs mainly include cryopreservation, lyophilization and spray drying (156). -80°C is considered the optimal temperature with minimal impact on the morphology and content of exosomes (156).

How long manufactured EVs can be stored *in vitro* and *in vivo* stability after storage remain unknown. At present, there are no consistent reports on related studies. Even though there are some limitations to the clinical application of EVs, there are still many clinical trials of EVs (146). At present, the ability of EVs to reverse bone imbalance, stimulate osteoblast activity, inhibit osteoclast activity, and deliver siRNA to regulate the osteoporosis microenvironment in osteoporosis has been widely reported, but the reports in Evs in DOP are still insufficient. We believe that EVs will make a breakthrough in the field of DOP in the near future.

## 5 Prospect

EVS has demonstrated its potential in tissue engineering and regenerative medicine in preclinical studies in recent years. EVs have low immunogenicity, biological histocompatibility, and can avoid the side effects of cell therapy. EVS is capable of being chemically modified to be targeted. At the same time, EVs can also act as carriers to release their contents into target cells. Preclinical studies have demonstrated that EVs can reduce the incidence of DOP and reduce the complications of fractures in DOP. Although there are few reports of preclinical studies on the use of EVs in DOP, there have been more clinical trials of EVs in other fields. At present, the problems faced by EVS have been recognized by researchers, and many improvement solutions have been designed. We believe that as people’s awareness of EVs continues to rise, there will definitely be a breakthrough in the application of EVs in DOP.
